# Embarrassment and Shame in People With Parkinson's Disease: A New Tool for Self-Assessment

**DOI:** 10.3389/fneur.2020.00779

**Published:** 2020-07-31

**Authors:** Vanessa Fleury, Sabina Catalano Chiuvé, Maria João Forjaz, Mariagrazia Di Marco, Maria Messe, Ines Debove, Julio Angulo, Gun-Marie Hariz, Pierre R. Burkhard, Pablo Martinez-Martin, Carmen Rodriguez-Blazquez, Paul Krack

**Affiliations:** ^1^Faculty of Medicine, University of Geneva, Geneva, Switzerland; ^2^Division of Neurology, Geneva University Hospitals, Geneva, Switzerland; ^3^National Centre of Epidemiology, Carlos III Institute of Health, REDISSEC, Madrid, Spain; ^4^Center for Networked Biomedical Research in Neurodegenerative Diseases (CIBERNED), Carlos III Institute of Health, Madrid, Spain; ^5^Clinical Investigation Unit, Geneva University Hospitals, Geneva, Switzerland; ^6^Department of Neurology, Inselspital, University Hospital Bern, University of Bern, Bern, Switzerland; ^7^Morningview Place, Lake Oswego, OR, United States; ^8^Member, Persons With Parkinson's Advisory Council, Parkinson Foundation, Miami, FL, United States; ^9^Member, Program Design Committee 2019 World Parkinson's Congress, World Parkinson's Coalition, New York, NY, United States; ^10^Department of Clinical Science, Neuroscience, Umeå University, Umeå, Sweden

**Keywords:** parkinson's disease, shame, embarrassment, questionnaire, non-motor symptoms

## Abstract

Shame and embarrassment related to Parkinson's disease (PD) are rarely addressed in clinical practice nor studied in neuroscience research, partly because no specific tool exists to detect them in PD.

**Objective:** To develop a self-applied assessment tool of shame and embarrassment specifically related to PD or its treatment, to promptly identify the presence and severity of these two emotions in PD.

**Methods:** Identification and selection of relevant items were obtained from the collection of PD patients' opinions during support groups and interviews. Several further items were added following a literature review. Subsequently, a two-phase pilot study was performed for identification of ambiguous items and omissions, and to obtain preliminary data on acceptability, reliability, validity and relevance of the new scale (SPARK).

**Results:** A total of 105 PD patients were enrolled in the study. Embarrassment was reported in 85% of patients, while shame was present in 26%. Fifteen percent of patients did not describe any shame or embarrassment. On average, the intensity of these two emotions was low with a marked floor effect in SPARK items and subscales. However, SPARK total score inter-individual variability was important (range 1–84 out of 99). Acceptability and quality of data were satisfactory with no floor or ceiling effects (2.9% each) or missing data. Internal consistency (Cronbach's alpha) was 0.94 for total score and 0.73–0.87 for subscales. The scale correlated ≥0.60 with instruments measuring related constructs. Content validity was satisfactory. SPARK total score strongly correlated with impaired health-related quality of life (r_S_ = 0.81), the propensity to feel embarrassed or ashamed (r_S_ = 0.68 and 0.66, respectively), and anxiety (r_S_ = 0.72) and depression (r_S_ = 0.63) levels. Moderate to high correlations were observed between SPARK total score and apathy (r_S_ = 0.46) and a more pronounced personality trait directed toward harm avoidance (r_S_ = 0.46). No significant differences in SPARK scores were found by sex, education level, PD duration, Hoehn and Yahr stages or PD phenotype.

**Conclusion:** Preliminary analysis of psychometric properties suggests that SPARK could be an acceptable and reliable instrument for assessing shame and embarrassment in PD. SPARK could help healthcare professionals to identify and characterize PD-induced shame and embarrassment.

## Introduction

Patients affected with Parkinson's disease (PD) perceive non-motor symptoms as serious challenges and barriers to a satisfying quality of life (1). PD-related shame and embarrassment are rarely addressed in clinical practice nor studied in neuroscience research (2). The prevalence of shame and embarrassment in PD is unknown and no specific tool exists to detect and measure them in PD.

Shame and embarrassment are two negative self-conscious emotions associated with painful states, where the self (i.e., the affective representation of one's identity) is focal in attention. The individual believes that she/he has failed to meet appropriate standards of conduct, and thinks that she/he has done so in the eyes of others. No consensus has been reached on how shame and embarrassment differ (3). Intuitively, for English speakers at least, shame and embarrassment are members of the same family and the differences between the two are subtle. The establishment of explicit differential criteria to distinguish shame from embarrassment has proven difficult in the literature. Shame is psychologically more challenging than embarrassment, marked by intensely painful negative self-evaluation commonly exhibited by an individual upon realizing that she/he has committed an offense or violated an important (usually social) norm. Shame is more long-lasting and produces more damage to self-esteem. Shame is also associated with a more serious breach of fundamental norms or rules. Upon contemplating the transgression, the individual concludes that she/he is incapable, worthless, fundamentally flawed, reprehensible, and worthy of contempt. Whereas, embarrassment is about minor transgressions or failures in role enactments or failure in one's ability to present her/himself to others in an ideal manner. Embarrassment is associated with a motivational response directed toward the preservation of one's social reputation, rather than a concern for others' well-being and a need to make amends, as in guilt, or with a concern for oneself with a need to hide as in shame (4).

Shame and embarrassment in PD may emerge from different sources: (1) PD symptoms, especially visible motor symptoms but also non-motor symptoms; (2) increasing physical dependence and need for help induced by PD; and (3) deteriorated body image (2). Consequences of PD-related shame on health-related quality of life are probably important but have not been studied in detail. Consequently, shame and embarrassment should be actively explored and addressed in patients affected with PD.

To do so, a specific tool to detect and measure these emotions in PD is needed. We therefore created a self-applied questionnaire rating shame and embarrassment specifically induced by PD or its treatment, to promptly identify the presence and severity of these emotions in PD patients as well as to better understand what clinically promotes these emotions. The objective of this pilot study is to describe the development process of this rating scale, including its conception and the analysis of its relevance and adequateness to the target population as well as its psychometric properties.

## Materials and Methods

The study was approved by the Geneva Ethics Committee. All participants gave their informed written consent.

### Identification and Selection of Items of Interest (Phase 1)

Identification of relevant items was based on a set of opinions and perspectives expressed by 44 PD patients during support groups and informal interview. The inclusion criteria for participants was a diagnosis of PD. The only exclusion criteria was the presence of dementia. The content of the expressed views was subsequently analyzed and reduced to a set of qualitative themes or meaning units. This preliminary phase of the study was from our point of view an essential component, as it allowed us to better understand shame and embarrassment related to the disease in PD patients. In addition, a comprehensive review of the literature on shame and stigmatization in PD was carried out. It revealed that the utterances expressed by patients were, generally, on the mark. Following further understanding obtained from this review process, new items were added to the emerging scale. Extra items were implemented from two scales: 2 items from the PDQ-39 (5) and 6 items from the Stigma Scale for Chronic Illness 8-item version (6). Data obtained during phase 1 provided the construction of a preliminary scale including 26 items. Responses reflected a scale of intensity (from 0 to 3: 0 = not at all, 1 = a little, 2 = moderately, 3 = very much). The scale was called Shame and embarrassment in Parkinson's disease (SPARK).

### Construction of the First Draft of the Scale and Pre-testing (Phase 2)

The preliminary SPARK scale was applied to 26 patients with a diagnosis of PD based on the United Kingdom Parkinson's Disease Society Brain Bank criteria (7). Patients were recruited from the Neurology Department of the Geneva University Hospital. PD patients with any kind of dementia (including mild and moderate) defined by a Montreal cognitive assessment (MOCA) score (8) <26/30 were excluded. A cognitive debriefing questionnaire was administered after completion of the SPARK scale, asking patients about their opinions on the relevance of the subject for their medical follow-up, length of the questionnaire, simplicity to respond, embarrassment with any item, omissions and global view. This was done to identify ambiguities, redundancies and omissions as well as to obtain preliminary data of acceptability and relevance of the subject. This questionnaire consists of 8 items with two possible answers (yes or no). A space for text where subjects could express their opinion was also available for each question.

### Reformulation and Construction of the Second Version of the Scale

The preliminary scale was adapted after the analysis of the cognitive debriefing questionnaire. The new scale was reviewed by five experts in PD and four experts in questionnaire validation. A second version of the SPARK questionnaire was created, with 33 items grouped into 6 subscales: (1) Shame and embarrassment arising from PD symptoms (items 1–5, 8, 11–16, 20); (2) Shame and embarrassment arising from the increasing physical dependence and need for help induced by PD (items 7, 10, 18); (3) Shame and embarrassment arising from the deteriorated body image (items 6, 9, 17, 19, 21); (4) Consequence of related shame and embarrassment on patient's self-esteem (items 22, 25, 26); (5) Stigmatization (items 23, 24, 27–31); (6) Type of emotion (item 32 for embarrassment, item 33 for shame). A summary score was calculated by adding up all individual item scores, for a maximum of 99 points. The self-assessment SPARK questionnaire takes ~5 min to perform.

### Testing of the Second Version of the Scale (Phase 3)

Thirty-five PD patients with no dementia were enrolled. A neurological assessment was performed including a brief medical history aimed to determine PD duration, stage of the disease established by Hoehn and Yahr scale (9), levodopa equivalent daily dose (LEDD) (10) and educational level, as well as a motor assessment including a MDS-UPDRS part 3 (11), the determination of the type of PD phenotype (12) and the level of dyskinesia using the Marconi Dyskinesia Rating Scale (13). The previously described SPARK debriefing questionnaire used during the pretesting phase was applied. Other questionnaires were also administered to assess psychobehavioral symptoms such as depression using the Beck Depression Inventory II (BDI-II) (14), anxiety with the State-Trait Anxiety Inventory for Adults (STAI) (15) and apathy with the Apathy scale (16). Personality dimensions were assessed with the Tridimensional Personality questionnaire (TPQ) (17). The impact on health-related quality of life was studied with a shorter version of the Parkinson's Disease Questionnaire (PDQ)-39, called the PDQ-8 (18, 19). To compare our results with two previously validated scales exploring the propensity to feel embarrassed or ashamed, we used the Personal Feelings Questionnaire (PFQ-2) (20–22) and the Embarrassment scale (21, 23).

### Construction of the Final Version of the Scale

**Figure 1:**
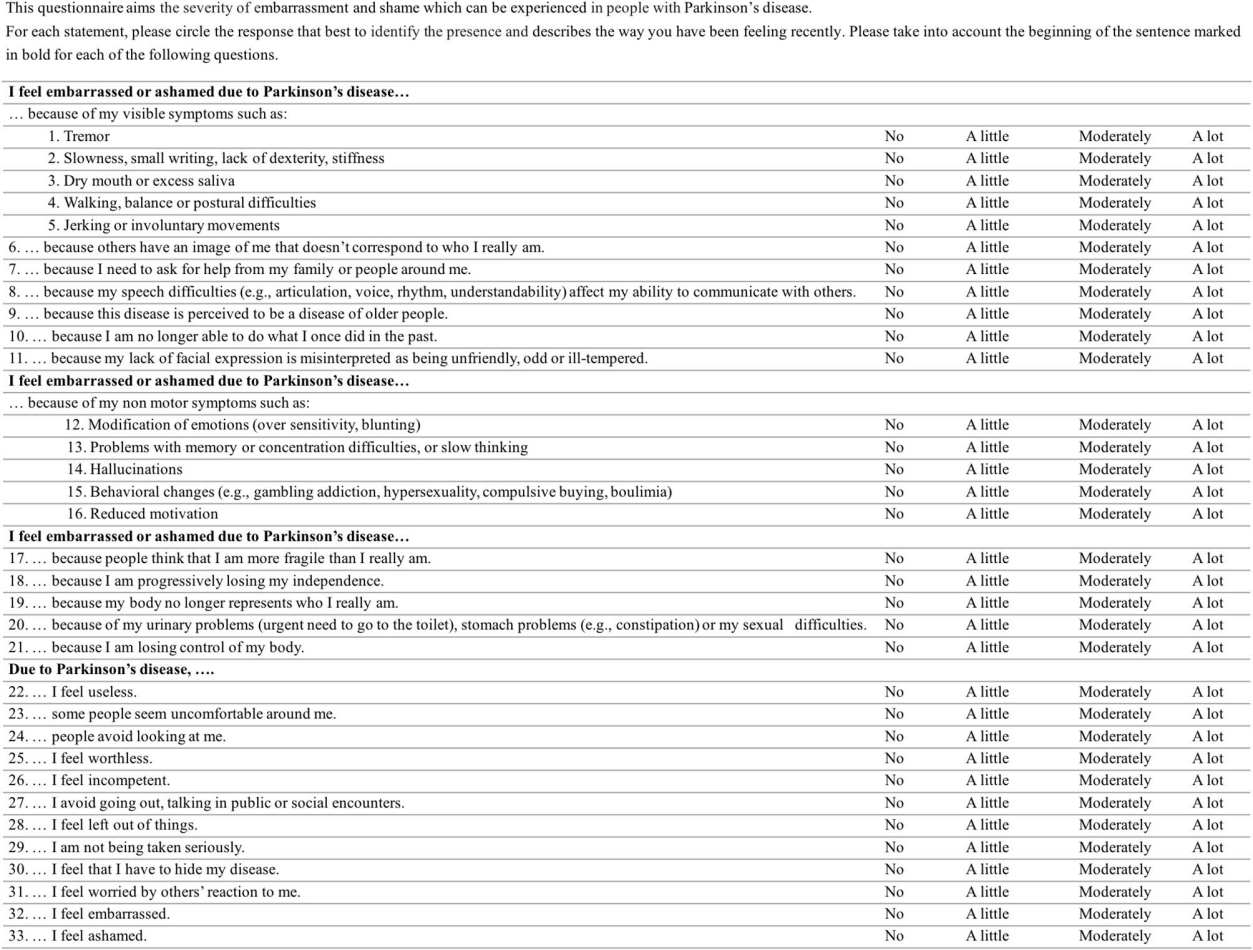
The SPARK questionnaire.

Patients' comments provided during the debriefing questionnaire were discussed between the authors. Comments judged relevant were used to create the final version of the SPARK scale (Figure 1 and Supplementary Data 1) that will be used in a future validation study.

### Data Analysis

Descriptive statistics of the sample characteristics and the applied rating scales were carried out. SPARK psychometric properties were studied only in the 35 PD patients who took part in phase 3 of the study. The following psychometric properties were analyzed, following the Classical Test Theory (CTT) (24):

- Data quality and acceptability (25, 26): missing data (standard criterion: <10%), fully computable data (criterion: >90%); distribution of scores, floor and ceiling effects (criterion: <15%) and skewness (criterion: between −1 and +1).
- Reliability in terms of internal consistency (27, 28): Cronbach's alpha (standard criterion: >0.70), inter-item correlation (criterion: 0.20–0.75), item homogeneity coefficient (criterion: >0.15) and corrected item-total correlation (criterion: ≥0.30).
- Validity. Three aspects of validity were assessed: convergent and known-groups validity (29), internal validity and content validity. For convergent validity, Spearman rank correlation (r_S_) was calculated between SPARK (total and subscale scores) and scores obtained with the two previously validated scales measuring shame and embarrassment as well as scores obtained with scales measuring depression, anxiety, apathy, type of personality and quality of life impact, constructs that theoretically should be related with the shame and embarrassment. A high correlation was established if the coefficient value r_S_ was ≥0.60 (30). A moderate to high correlation was considered if r_S_ was between 0.30 and 0.59. A moderate or weak was defined if r_S_ was <0.30. Known-group validity was tested by determining the differences in SPARK total and subscales scores with subgroups based on sex, level of education according to the International Standard Classification of Education, PD duration (by the median), Hoehn and Yahr (9) severity stage, and type of PD phenotype (using MDS-UPDRS scores and applying the formula as explained in Supplementary Table) (12). The Mann-Whitney test was utilized to determine the significance of the differences. Internal validity was assessed by means of the inter-correlation of domains, using Spearman's rank correlation coefficients (criterion, r_S_ = 0.30–0.70). For content validity, in addition to the input from experts opinion and literature review during the construction process, the qualitative evidence from the pre-testing debriefing questionnaire with patients was analyzed, to ensure that items in the scale were representative of the construct being measured (28). For the debriefing questionnaire, the frequency of yes/no responses was reported. The comments of the patients were analyzed descriptively to assess their opinions. All calculations were made using IBM SPSS version 25.0.

## Results

A total of 105 patients were enrolled in our study: 44 patients for phase 1, 26 for phase 2 and 35 for phase 3 of the pilot study. Demographic and clinical characteristics are reported in Table 1. Among the 61 patients who participated in phases 2 and 3 of the study, most patients (85%) were experiencing embarrassment, whereas shame concerned fewer patients (26%). When shame was present, embarrassment was always associated with it. Fifteen percent of patients did not describe any shame or embarrassment.

**Table 1:** Patients demographic and clinical characteristics for the three phases of the study.

	**Numbers**	**Median**	**Range (min-max)**
	**Pilot study 1**		
Number of participants (men)	44 (15)		
Age (years)		65	60–75
Disease duration in years		5.0	2–10
	**Pilot study 2**		
Number (men)	26 (16)		
Age (years)		64.6	39–82
Hoehn and Yahr (/4)		2	1.5–4
Motor score MDS-UPDRS III (/132)		18	5–40
MOCA (/30)		28	26–30
	**Pilot study 3**		
Number (men)	35 (15)		
Age (years)		67	43–77
Education level (I, II, ≥ III)	4 I, 12 II, 19 ≥ III		
Disease duration in years		8.7	1.9–19.1
Motor score MDS-UPDRS III (/132)		17	6–54
Dyskinesia score (/28)		2	0–10
Levodopa-equivalent daily dose (mg/day)		785	100–2,100
PD phenotype	10 TR, 24 AR, 1 Mixed		
MOCA (/30)		29	26–30
Hoehn and Yahr (/4)		2	1.5–4
PDQ-8 (/32)		9	0–27
Apathy scale (Starkstein) (/42)		9	3–23
Anxiety state score (STAI) (/80)		25	20–58
Anxiety trait score (STAI) (/80)		34	23–58
Depression score (BDI-II) (/63)		9	1–28

*BDI-II, Beck Depression Inventory II; Dyskinesia, Marconi Dyskinesia Rating scale; MDS-UPDRS motor score, motor scale of Movement Disorder Society Unified Parkinson's Disease Rating Scale; MOCA, MOntreal Cognitive Assessment; PD, Parkinson's disease; PD, phenotype: TR, tremor-dominant; AR, akinetic-rigid dominant; PDQ-8, Parkinson's Disease Questionnaire; Education level, Level I is defined as subjects who received a primary education, Level II a lower secondary education, and level 3 and above at least an upper secondary education*.

The phase 3 of our study analyzed SPARK's psychometric properties. The SPARK total mean score was 23.97 (standard deviation SD: 18.53; range: 1–84) (Table 2). SPARK total score presented a skewness of 1.50, with no floor or ceiling effects (2.9% each) or missing data. Responses to items covered the full range of scale scores (0–3) except for 3 items. Most subscales and items showed a marked floor effect (Table 2).

**Table 2:** Data quality and acceptability of SPARK.

	**N**	**Mean**	**Median**	**SD**	**Skewness**	**Min**	**Max**	**Floor effect %**	**Ceiling effect %**
Item SPARK1	35	0.89	1.00	0.90	0.49	0	3	42.90	2.90
Item SPARK2	35	1.26	1.00	0.98	0.44	0	3	22.90	14.30
Item SPARK3	35	0.46	0.00	0.74	1.76	0	3	65.70	2.90
Item SPARK4	35	1.09	1.00	1.07	0.59	0	3	37.10	14.30
Item SPARK5	35	0.86	0.00	1.11	0.97	0	3	54.30	14.30
Item SPARK 6	35	0.94	1.00	0.97	0.74	0	3	40.00	8.60
Item SPARK 7	35	0.60	0.00	0.81	1.23	0	3	57.10	2.90
Item SPARK 8	35	0.69	0.00	0.90	1.20	0	3	54.30	5.70
Item SPARK 9	35	0.74	0.00	1.12	1.21	0	3	62.90	14.30
Item SPARK 10	35	1.34	1.00	1.16	0.23	0	3	31.40	22.90
Item SPARK 11	35	0.86	0.00	1.11	0.97	0	3	54.30	14.30
Item SPARK 12	35	0.89	1.00	0.99	1.00	0	3	42.90	11.40
Item SPARK 13	35	0.77	1.00	0.91	1.23	0	3	45.70	8.60
Item SPARK 14	35	0.06	0.00	0.24	3.99	0	1	94.30	5.70
Item SPARK 15	35	0.46	0.00	0.92	1.82	0	3	77.10	5.70
Item SPARK 16	35	0.69	0.00	1.02	1.39	0	3	60.00	11.40
Item SPARK 17	35	0.60	0.00	0.91	1.65	0	3	60.00	8.60
Item SPARK 18	35	0.94	1.00	1.11	0.80	0	3	48.60	14.30
Item SPARK 19	35	0.86	1.00	0.97	1.11	0	3	42.90	11.40
Item SPARK 20	35	1.06	1.00	1.16	0.60	0	3	45.70	17.10
Item SPARK 21	35	0.77	0.00	0.97	0.90	0	3	54.30	5.70
Item SPARK 22	35	0.51	0.00	0.89	1.97	0	3	65.70	8.60
Item SPARK 23	35	0.40	0.00	0.60	1.26	0	2	65.70	5.70
Item SPARK 24	35	0.09	0.00	0.28	3.09	0	1	91.40	8.60
Item SPARK 25	35	0.34	0.00	0.84	2.44	0	3	82.90	5.70
Item SPARK 26	35	0.63	0.00	0.97	1.65	0	3	60.00	11.40
Item SPARK 27	35	1.00	1.00	1.16	0.71	0	3	48.60	17.10
Item SPARK 28	35	0.54	0.00	0.85	1.37	0	3	65.70	2.90
Item SPARK 29	35	0.29	0.00	0.67	2.75	0	3	80.00	2.90
Item SPARK 30	35	0.77	0.00	1.06	1.12	0	3	57.10	11.40
Item SPARK 31	35	0.71	0.00	1.07	1.22	0	3	62.90	11.40
Item SPARK 32 Embarrassment	35	1.34	1.00	0.87	0.37	0	3	14.30	11.40
Item SPARK 33 Shame	35	0.54	0.00	0.95	1.72	0	3	68.60	8.60
Subscale SPARK PD symptoms	35	10.00	9.00	7.21	1.34	1	30	5.70	2.90
Subscale SPARK Physical dependence	35	3.91	3.00	3.84	0.66	0	9	25.70	2.90
Subscale SPARK Body image deterioration	35	2.89	3.00	2.52	1.32	0	15	17.10	2.90
Subscale SPARK Self-esteem	35	1.49	1.00	2.42	2.17	0	9	48.60	5.70
Subscale SPARK Stigmatization	35	3.80	3.00	3.94	0.96	0	15	28.60	2.90
SPARK **Total (/99)**	**35**	**23.97**	**19.00**	**18.53**	**1.50**	**1**	**84**	**2.90**	**2.90**

*N, number of items in the questionnaire; SD, standard deviation*.

Regarding internal consistency (Table 3), Cronbach's alpha ranged from 0.73 (Physical dependence subscale) to 0.87 (subscale Self-esteem), with a value of 0.94 for the total score. Item homogeneity coefficient ranged from 0.29 (PD symptoms subscale) to 0.70 (Self-esteem subscale). Most items (except items 1, 3 and 24) showed an item-total corrected correlation >0.40.

**Table 3:** Internal consistency of SPARK.

**Subscales**	**Item-total corrected correlation**	**Cronbach's Alpha**	**Inter-item correlation**	**Item homogeneity**
PD symptoms	0.01–0.71	0.84	−0.36–0.73	0.29
Physical dependence	0.44–0.71	0.73	0.30–0.63	0.47
Body image	0.58–0.71	0.83	0.41–0.80	0.50
Self-esteem	0.71–0.85	0.87	0.59–0.77	0.70
Stigmatization	0.05–0.62	0.77	−0.13–0.70	0.32

SPARK total score strongly correlated with PDQ-8 (r_S_ = 0.81), PFQ-2 total and Shame subscale (r_S_ = 0.66 and 0.69, respectively), Embarrassment scale (r_S_=0.68), STAI State and Trait (r_S_ = 0.62 and 0.72, respectively) and BDI-II (r_S_ = 0.63) (Table 4). Moderate to high correlations were observed between SPARK total score and Apathy scale (r_S_ = 0.46) and TPQ Harm avoidance (r_S_ = 0.46). A negative weak to moderate correlation was observed between SPARK total score and age (r_S_ = −0.37). PD duration, LEDD, motor score and type of PD phenotype showed weak to moderate correlations with SPARK. All SPARK subscales significantly correlated with PDQ-8 (r_S_ ≥ 0.60). SPARK subscales PD Symptoms and Body image strongly correlated (r_S_ = 0.68 and 0.73, respectively) with PFQ-2 Shame subscale; and PD Symptoms and Stigma strongly correlated (r_S_ = 0.63 and 0.68, respectively) with the Embarrassment scale. PD duration, LEDD and MDS-UPDRS part 3 showed weak correlations with SPARK.

**Table 4:** Convergent validity of SPARK scale and subscales.

	**PD Symptoms**	**Physical dependence**	**Body image**	**Self- esteem**	**Stigma**	**Item 32 Embarr**.	**Item 33 Shame**	**SPARK TOTAL**
Age	−0.33	−0.27	−0.19	−0.23	−0.36*	−0.34*	−0.45**	−0.37*
Duration of PD	0.20	−0.09	0.22	−0.03	0.03	−0.13	−0.33	0.10
LEDD	0.27	−0.03	0.18	0.00	0.08	−0.12	0.04	0.11
MOCA	−0.23	−0.20	−0.22	−0.03	0.15	−0.00	0.31	−0.14
MDS-UPDRS 3	0.03	0.05	0.13	0.10	0.16	0.09	−0.02	0.10
Tremor score	−0.31	0.12	−0.14	−0.09	−0.32	−0.02	−0.05	−0.21
Akinetic-rigid score	0.19	0.06	0.22	0.20	0.31	0.08	0.02	0.23
PIGD score	−0.12	−0.01	0.06	−0.17	−0.20	−0.15	−0.28	−0.09
Apathy scale	0.39*	0.29	0.26	0.59**	0.45**	0.22	0.25	0.46**
PDQ-8 total	0.75**	0.64**	0.68**	0.60**	0.69**	0.47**	0.41*	0.81**
PFQ-2 total	0.62**	0.50**	0.63**	0.42*	0.53**	0.41*	0.40*	0.66**
PFQ-2 shame	0.68**	0.54**	0.73**	0.43*	0.52**	0.41*	0.26	0.69**
PFQ-2 guilt	0.40*	0.37*	0.36*	0.32	0.43*	0.34*	0.51**	0.47**
Embarrassment scale	0.63**	0.46**	0.59**	0.54**	0.68**	0.38*	0.22	0.68**
TPQ novelty seeking	0.32	0.15	0.32	0.11	0.21	−0.10	0.06	0.29
TPQ Harm avoidance	0.37*	0.44**	0.38*	0.60**	0.36*	0.20	0.24	0.46**
TPQ reward dependence	0.22	0.36*	0.28	0.14	−0.10	0.09	0.06	0.25
STAI state	0.55**	0.53**	0.43*	0.62**	0.46**	0.28	0.39*	0.62**
STAI trait	0.57**	0.64**	0.59**	0.61**	0.64**	0.48**	0.36*	0.72**
BDI-II total	0.56**	0.53**	0.43**	0.48**	0.52**	0.35*	0.47**	0.63**

*Scores are expressed using Spearman rank correlation coefficient value (r_S_). A score r_S_ ≥ 0.60 is considered to have a high correlation level. A score between 0.30 and 0.59 is considered to have a moderate to high correlation level. A score <0.30 is considered to have a moderate to weak correlation level*.

*p < 0.05; **p < 0.01. p refers to the significance level of the correlation coefficients.

*BDI-II, Beck Depression Inventory II; Embarr, embarrassment; LEDD, levodopa equivalent daily dose; MDS-UPDRS, Movement Disorder Society Unified Parkinson's Disease Rating Scale; MOCA, Montreal Cognitive Assessment; PD, Parkinson's disease; PDQ-8, Parkinson's Disease Questionnaire (Health-related quality of life), 8 items; PQF-2, Personal Feelings Questionnaire (shame and embarrassment scale); TPQ, Tridimensional Personality Questionnaire; STAI, State-Trait Anxiety Inventory*.

Regarding known-groups validity (Supplementary Data 2), SPARK total and subscales scores did not present significant differences by sex, education level, PD duration, Hoehn and Yahr severity stages or PD phenotype.

Regarding internal validity, SPARK subscales correlated from 0.31 to 0.74 between them (Table 5). In terms of content validity, patients' responses to the debriefing questionnaire (Table 6) demonstrated that >85% of the group found the scale to be relevant to their current situation, helpful for their healthcare professionals to understand their current state, understandable and with adequate length. Questions were described as embarrassing or difficult to answer only by 8.6 and 11.4% of the sample, respectively. Thirty-two percent of patients made comments. See Supplementary Data 3 for a summary of patients' comments. Some items were consequently modified and some subitems were added in order to capture the topic as comprehensively as possible. Comments on embarrassment and shame induced by sleep disturbances such as daytime sleepiness and acting out dreams were not included in the final version of the scale because this comment was made by a single patient.

**Table 5:** Internal validity.

	**PD symptoms**	**Physical dependence**	**Body image**	**Self Esteem**	**Stigma**	**Item 32 embarrassment**
Physical dependence	0.59**					
Body image	0.71**	0.74**				
Self Esteem	0.60**	0.56**	0.49**			
STIGMA	0.61**	0.47**	0.59**	0.70**		
Item 32 Embarrassment	0.38*	0.58**	0.47**	0.45**	0.57**	
Item 33 Shame	0.48**	0.34*	0.31	0.43**	0.53**	0.48**

Standard: r_S_ > 0.50; * p < 0.05; ** p < 0.01.

**Table 6:** Responses to the debriefing questionnaire about SPARK.

	**Answer**	***N***	**%**
Relevance for the patient's current situation	No	5	14.3
	Yes	30	85.7
Helpfulness for their healthcare professionals to understand the patient's current situation	No	1	2.9
	Yes	34	97.1
Good understandability	No	0	0.0
	Yes	35	100.0
Missing aspects	No	24	68.6
	Yes	11	31.4
Lenght	No	34	97.1
	Yes	1	2.9
Embarrassing questions	No	32	91.4
	Yes	3	8.6
Difficulty to answer questions	No	31	88.6
	Yes	4	11.4
Comments	No	22	62.9
	Yes	13	37.1

*N, number of patients*.

## Discussion

SPARK is a new self-administered questionnaire assessing shame and embarrassment induced by PD. The aim of our study was to show how SPARK was conceived and designed. In addition, some psychometric properties have been tested to orient the developers toward potential problems with the current structure. Preliminary analyses of the psychometric properties suggest that SPARK could be an acceptable and reliable instrument for assessing shame and embarrassment in PD. Higher scores of shame and embarrassment were related to impaired health-related quality of life and higher levels of depression and anxiety. Consequently, PD-related embarrassment and shame probably deserve our attention. SPARK could be a useful tool for healthcare professionals and researchers to identify and rate these two negative emotions, as well as to better understand what clinically promotes these two painful and disruptive emotions.

Regarding the psychometric analysis, SPARK had a satisfactory acceptability and data quality with no missing data, due to good procedures during data collection. Internal consistency and internal validity were acceptable, suggesting that the scores of our instrument were an adequate reflection of the dimensionality of the construct (embarrassment and shame) that we thought to measure. Content validity was very satisfactory, with the vast majority of patients thinking that the questionnaire was relevant to their current situation and could be helpful for their healthcare professional for their follow-up. SPARK was judged by patients as easily understandable and of adequate length, taking about 5 min to complete. The content validity was excellent, probably due to the SPARK construction and testing process which involved a collaborative effort with multiple exchanges between PD patients and healthcare professionals specialized in PD.

In terms of the frequency of shame and embarrassment induced by PD, most of our patients (85%) were experiencing embarrassment whereas shame concerned far fewer patients (26%). When shame was present, it was associated with embarrassment in 100% of cases. Our results argue for the fact that embarrassment and shame are two closely related self-conscious emotions belonging to the same continuum of emotion, varying on a range of factors such as intensity, public exposure and physical reaction (31). To the best of our knowledge, the exact prevalence of the shame and embarrassment in PD is unknown and our percentages would have to be checked in a larger sample. Parkinson's UK, a patients' association, found that 41% of PD patients reported experiencing discrimination because of PD, including some experiences of misinterpretation of symptoms or verbal abuse in public (32).

In terms of the intensity of shame and embarrassment, SPARK total score had a mean of 24 out of 99 with a wide range of scores (1–84) showing that the severity of the shame and embarrassment varied greatly among patients. Floor effect in SPARK items and subscales indicated that most patients showed low levels of shame and embarrassment. However, SPARK scores were associated with a lower level of health-related quality of life, as well as with higher levels of depression and anxiety. Shame and embarrassment may contribute to psychological difficulties such as personal distress, self-identity alteration, social isolation, depression, and social anxiety (33–36). The impact of shame and embarrassment on patients' quality of life might be exacerbated by the fact that patients do not talk about this feeling because it is a taboo subject (37). Many PD patients do not spontaneously discuss these experiences with their relatives or their neurologist because, ironically, they think that it is considered embarrassing or shameful to talk about one's embarrassment or the sources of one's shame (2).

The wide range of SPARK scores among patients probably reflects the inter-individual variability of the experience of shame and embarrassment. These two emotions vary depending on self-awareness, personality traits, level of self-esteem and self-blame, and culture (38–41). Our results are in accordance with this assumption, whereby higher SPARK scores were related to the personality propensity to feel embarrassed or ashamed and with a personality trait directed toward harm avoidance.

The role of PD neuropathology itself in the experience of shame and embarrassment is unknown. According to our study, an indirect and a direct role of PD are probable. An indirect role is probable through symptoms caused by PD as well as the increasing physical dependence and the deteriorated body image. However, a direct role of PD on the emotional experience might also be associated, but remains to be demonstrated. PD is secondary to neurodegeneration involving predominantly dopaminergic neurons (42). Higher SPARK scores were related to a more pronounced personality variant toward harm avoidance, whereas SPARK scores were not linked with the two other personality dimensions defined by Cloninger's biosocial model of personality (novelty seeking and reward dependence) (43). The harm avoidance dimension is characterized by a tendency to respond intensely to signals of aversive stimuli, thereby learning to inhibit behavior to avoid punishment, novelty and frustrative non rewarding situations. Individuals with higher levels of harm avoidance show anticipatory worry, fear of uncertainty, shyness with strangers as well as fatigability and asthenia. Yet harm avoidance has been linked with hypodopaminergic behaviors such as apathy, depression, anxiety, irritability, and hyperemotionality (44–46) as well as with PD (47). In addition, SPARK scores were strongly related with higher levels of depression and anxiety, and moderately associated with the level of apathy. We hypothesized a dopaminergic modulation to embarrassment and shame in PD. We expect that shame and embarrassment would decrease in the case of hyperdopaminergia (euphoria, hyperactivity, hypomania, and impulse control disorders) when also depression, anxiety, apathy and harm avoidance largely disappear. This assumption remains to be elucidated.

No relationship was found between the intensity of PD-induced shame and embarrassment and PD duration and the severity of motor symptoms. These results might suggest that other factors might be involved in the intensity of the shame and embarrassment. A longitudinal study investigating the evolution of SPARK scores depending on the phase of PD would be interesting. Our hypothesis is that the intensity of shame and embarrassment might be higher before the dopaminergic replacement therapy is introduced and during the early post-diagnosis phase when the patient is learning to adapt to her/his disease. Another hypothesis would be that shame and embarrassment are little related with motor symptoms as compared with neuropsychiatric symptoms.

Nevertheless, our scale contains several limitations. Three items presented low item-total corrected correlations with their respective subscales. Two pairs of items showed high inter-item correlation which might suggest redundancy. This aspect will be checked during the validation study in a larger sample. As pointed out by the debriefing questionnaire, some aspects contributing to shame and embarrassment were missing. Some new items were consequently added (stiffness, dysarthria, dry mouth, posture difficulties) in order to capture the topic as comprehensively as possible. The final version of SPARK will be utilized during the future validation study. As mentioned by several patients and because of the type of rating scale that we chose, SPARK measures the intensity of shame and embarrassment but not the frequency of the occurrence of these two emotions. SPARK also does not differentiate clearly if shame and embarrassment are internal or external. Internal shame or embarrassment describes the negative evaluation a person applied to her/himself whereas external shame or embarrassment relates to the evaluation of what the person believes others think about her/him i.e. the distressing awareness that “I think others view me negatively” (48). The amount of psychosocial support received by patients was not able to be measured. It is however data that may well influence PD-induced shame and embarrassment and in this sense, should be taken into account in future studies. Finally, SPARK could encounter difficulties at a linguistic level in non-English or non-French speaking countries. Indeed, the distinction between shame and embarrassment may not be obvious depending on the language. In Spanish for example, the distinction between shame and embarrassment does not exist in the common (everyday life) language. For this reason, we chose to combine embarrassment and shame as a single combined score. Future studies should also address the role of culture on shame and embarrassment in PD. How emotions are understood and expressed varies across cultures (40, 41). Some social groups view the self in individualistic psychological terms as a self that is bounded, separate from others. Shame and embarrassment are then perceived as a psychological event occurring inside an individual. Meanwhile, other cultures favor a collectivist conception wherein shame and embarrassment are emotions that happen interpersonally, outside, between people (39). The appraisal of how shame and embarrassment are felt and expressed, as well as the responsibility for resolving them, also varies (38). International studies are needed, with a more diverse sample of PD patients in order to explore cultural differences regarding the embarrassment and shame by a formal study. Finally, our sample size was relatively small.

In conclusion, the SPARK scale could be a reliable questionnaire which promptly measures the severity of shame and embarrassment specifically induced in patients in the context of PD. A validation study would be useful to confirm this assumption. The availability of this rating scale could raise awareness on these two emotions in PD. The SPARK scale could help healthcare professionals to identify the problem of shame and embarrassment affecting PD patients and to better understand what clinically promotes these two emotions. Our study demonstrated that PD-associated embarrassment is extremely frequent. Shame and embarrassment were associated with a lower level of health-related quality of life, as well as with higher levels of depression and anxiety. As such, PD-related shame and embarrassment deserve our attention. Further studies are needed to deepen the understanding of the subject, such as studies exploring clinical, cultural or socioeconomic factors influencing these two painful emotions. The clinical implication of this score system could be important especially in patients who score high on SPARK total score and shame sub-item as shame probably contributes to psychological difficulties such as personal distress, depression, suicidal ideation, and social encounter avoidance. A high SPARK score should alert healthcare professionals to the potential presence of psychological difficulties. The SPARK questionnaire could therefore help healthcare professionals to implement psychological support to patients' management in a timely fashion in order to help patients cope with their disease. Cognitive-behavioral intervention strategies such as systematic desensitization, role playing, thought stopping, disputing the inner critic, identification of irrational thinking and dysfunctional cognitive schemas could be clinically beneficial for these 2 emotions (49, 50). The SPARK scale could also help indirectly researchers to better understand the biological role of monoaminergic neurotransmitter depletion in these negative emotions. Understanding the biology behind shame and embarrassment could allow more targeted pharmacological management in addition to enhancing coping strategies.

## Data Availability Statement

All datasets presented in this study are included in the article/Supplementary Material.

## Ethics Statement

The studies involving human participants were reviewed and approved by Geneva Ethics Committee (SwissEthics). The patients/participants provided their written informed consent to participate in this study.

## Author Contributions

VF, SC, MF, MM, ID, G-MH, PM-M, CR-B, and PK: conception and design of the study. VF, SC, MF, MM, JA, PB, and CR-B: acquisition and analysis of data. VF, SC, MF, MD, MM, ID, G-MH, JA, PB, PM-M, CR-B, and PK: writing and review of the text and preparing tables and figures. All authors contributed to the article and approved the submitted version.

## Conflict of Interest

The authors declare that the research was conducted in the absence of any commercial or financial relationships that could be construed as a potential conflict of interest.

## Abbreviations

BDI-II: Beck Depression Inventory II
LEDD: levodopa equivalent daily dose
MDS-UPDRS motor score: motor scale of Movement Disorder Society Unified Parkinson's Disease Rating Scale
MOCA: MOntreal Cognitive Assessment
PD: Parkinson's disease
PD: phenotype
TR: tremor dominant
AR: akinetorigid form
PDQ-8: Parkinson's Disease Questionnaire 8 items (Health-related quality of life)
PFQ-2: Personal Feelings Questionnaire (shame and embarrassment scale)
SD: standard deviation
STAI: State-Trait Anxiety Inventory
SPARK: Shame and embarrassment in PARKinson's disease questionnaire
TPQ: Tridimensional Personality Questionnaire.
