# Supplementary material for: Embarrassment and Shame in People With Parkinson's Disease: A New Tool for Self-Assessment
Source: Front Neurol. 2020 Jul 31;11:779. doi: 10.3389/fneur.2020.00779 (PMC7411180; doi:10.3389/fneur.2020.00779)
Supplement: Supplementary file 1 [file Table_1.DOCX]

**Instructions for administration and scoring of the questionnaire SPARK**

Embarrassment and shame are negative emotions occurring in situations where one assesses and judges oneself. These emotions arise when a person thinks she/he has breached a norm, or while she/he has the impression that her/his own internal standards or the external standards of the society are not met.

Shame is a painful emotion involving a negative evaluation of the whole self. It is associated with a sense of weakness and a lack of self-esteem.

Embarrassment occurs in situations where failures or transgressions are less important. It is an emotion occurring suddenly because of a blunder or clumsy behavior that undermines the esteem that others have for us.

The scoring system is as follows:

No = 0 point; A little = 1 point; Moderately = 2 points; A lot = 3 points.

**Total score:** /99

**Subscales:**

1. Embarrassment and shame arising from Parkinson’s disease symptoms:

Items 1, 2, 3, 4, 5, 8, 11, 12, 13, 14, 15, 16, 20

Subscore: /39

1. Embarrassment and shame arising from the increasing physical dependence and need for help induced by Parkinson’s disease:

Items 7, 10, 18

Subscore: /9

1. Embarrassment and shame arising from a deteriorated body image:

Items 6, 9, 17, 19, 21

Subscore: /15

1. Consequences of Parkinson’s disease on patients’ self-esteem:

Items 22, 25, 26

Subscore: /9

1. Stigmatization

Items 23, 24, 27, 28, 29, 30, 31:

Subscore: /21

1. Types and intensity of self-evaluative negative emotion:

Item 32 for embarrassment: /3

Item 33 for shame: /3
