# Supplementary material for: Embarrassment and Shame in People With Parkinson's Disease: A New Tool for Self-Assessment
Source: Front Neurol. 2020 Jul 31;11:779. doi: 10.3389/fneur.2020.00779 (PMC7411180; doi:10.3389/fneur.2020.00779)
Supplement: Supplementary file 2 [file Table_2.DOCX]

**Supplementary Table.** Known-groups validity

By sex

| SPARK | | N | Mean | SD | Min | Max | p* |
| --- | --- | --- | --- | --- | --- | --- | --- |
| PD SYMPTOMS | men | 15 | 11.33 | 7.77 | 1 | 30 | 0.26 |
|  | women | 20 | 9.00 | 6.79 | 1 | 29 |  |
| PHYSICAL DEPENDENCE | men | 15 | 2.93 | 2.60 | 0 | 9 | 0.87 |
|  | women | 20 | 2.85 | 2.52 | 0 | 8 |  |
| BODY IMAGE | men | 15 | 4.27 | 4.45 | 0 | 15 | 0.91 |
|  | women | 20 | 3.65 | 3.41 | 0 | 14 |  |
| SELF ESTEEM | men | 15 | 2.07 | 3.01 | 0 | 9 | 0.23 |
|  | women | 20 | 1.05 | 1.82 | 0 | 6 |  |
| STIGMA | men | 15 | 5.20 | 4.87 | 0 | 15 | 0.23 |
|  | women | 20 | 2.75 | 2.75 | 0 | 9 |  |
| Item 32 Embarrassment | men | 15 | 1.53 | 0.99 | 0 | 3 | 0.31 |
|  | women | 20 | 1.20 | 0.77 | 0 | 3 |  |
| Item 33 Shame | men | 15 | 0.67 | 1.11 | 0 | 3 | 0.70 |
|  | women | 20 | 0.45 | 0.83 | 0 | 3 |  |
| TOTAL | men | 15 | 28.00 | 21.54 | 1 | 84 | 0.30 |
|  | women | 20 | 20.95 | 15.83 | 2 | 72 |  |

* Mann-Whitney

By education level according to International Standard Classification of Education (ISCED) (I + II are in the same group due to the short size of group I)

| SPARK | | N | Mean | SD | Min | Max | p* |
| --- | --- | --- | --- | --- | --- | --- | --- |
| PD SYMPTOMS | I + II | 16 | 9.25 | 7.03 | 1 | 29 | 0.52 |
|  | ≥ III | 19 | 10.63 | 7.49 | 1 | 30 |  |
| PHYSICAL DEPENDENCE | I + II | 16 | 2.56 | 2.22 | 0 | 8 | 0.59 |
|  | ≥ III | 19 | 3.16 | 2.77 | 0 | 9 |  |
| BODY IMAGE | I + II | 16 | 4.13 | 4.29 | 0 | 14 | 0.91 |
|  | ≥ III | 19 | 3.74 | 3.52 | 0 | 15 |  |
| SELF ESTEEM | I + II | 16 | 1.00 | 1.55 | 0 | 6 | 0.59 |
|  | ≥ III | 19 | 1.89 | 2.94 | 0 | 9 |  |
| STIGMA | I + II | 16 | 3.44 | 3.91 | 0 | 11 | 0.61 |
|  | ≥ III | 19 | 4.11 | 4.04 | 0 | 15 |  |
| Item 32 Embarrassment | I + II | 16 | 1.25 | 0.86 | 0 | 3 | 0.45 |
|  | ≥ III | 19 | 1.42 | 0.90 | 0 | 3 |  |
| Item 33 Shame | I + II | 16 | 0.50 | 0.89 | 0 | 3 | 0.92 |
|  | ≥ III | 19 | 0.58 | 1.02 | 0 | 3 |  |
| TOTAL | I + II | 16 | 22.13 | 18.33 | 2 | 72 | 0.49 |
|  | ≥ III | 19 | 25.53 | 19.06 | 1 | 84 |  |

Level I is defined as subjects who received a primary education, Level II a lower secondary education, and level 3 and above at least an upper secondary education.

* Mann-Whitney

By PD duration (grouped by the median)

| SPARK | | N | Mean | SD | Min | Max | p* |
| --- | --- | --- | --- | --- | --- | --- | --- |
| PD SYMPTOMS | ≤ 8.7 yrs | 18 | 10.17 | 8.80 | 1 | 30 | 0.45 |
|  | 8.8+ yrs | 17 | 9.82 | 5.31 | 1 | 23 |  |
| PHYSICAL DEPENDENCE | ≤ 8.7 yrs | 18 | 3.22 | 2.92 | 0 | 9 | 0.58 |
|  | 8.8+ yrs | 17 | 2.53 | 2.03 | 0 | 7 |  |
| BODY IMAGE | ≤ 8.7 yrs | 18 | 3.61 | 4.49 | 0 | 15 | 0.23 |
|  | 8.8+ yrs | 17 | 4.24 | 3.11 | 0 | 10 |  |
| SELF ESTEEM | ≤ 8.7 yrs | 18 | 2.00 | 3.16 | 0 | 9 | 0.87 |
|  | 8.8+ yrs | 17 | 0.94 | 1.09 | 0 | 3 |  |
| STIGMA | ≤ 8.7 yrs | 18 | 4.00 | 4.41 | 0 | 15 | 0.84 |
|  | 8.8+ yrs | 17 | 3.59 | 3.50 | 0 | 11 |  |
| Item 32 Embarrassment | ≤ 8.7 yrs | 18 | 1.44 | 0.86 | 0 | 3 | 0.61 |
|  | 8.8+ yrs | 17 | 1.24 | 0.90 | 0 | 3 |  |
| Item 33 Shame | ≤ 8.7 yrs | 18 | 0.83 | 1.15 | 0 | 3 | 0.07 |
|  | 8.8+ yrs | 17 | 0.24 | 0.56 | 0 | 2 |  |
| TOTAL | ≤ 8.7 yrs | 18 | 25.28 | 22.74 | 2 | 84 | 0.78 |
|  | 8.8+ yrs | 17 | 22.59 | 13.29 | 1 | 47 |  |

* Mann-Whitney

By Hoehn and Yahr stages (grouped)

| SPARK | | N | Mean | SD | Min | Max | p* |
| --- | --- | --- | --- | --- | --- | --- | --- |
| PD SYMPTOMS | Mild HY (1-2-2.5) | 28 | 9.79 | 7.4 | 1 | 30 | 0.48 |
|  | Moderate-severe HY (3-4) | 7 | 10.86 | 6.59 | 3 | 23 |  |
| PHYSICAL DEPENDENCE | Mild HY (1-2-2.5) | 28 | 2.75 | 2.67 | 0 | 9 | 0.39 |
|  | Moderate-severe HY (3-4) | 7 | 3.43 | 1.81 | 2 | 7 |  |
| BODY IMAGE | Mild HY (1-2-2.5) | 28 | 3.54 | 3.92 | 0 | 15 | 0.11 |
|  | Moderate-severe HY (3-4) | 7 | 5.43 | 3.31 | 0 | 10 |  |
| SELF ESTEEM | Mild HY (1-2-2.5) | 28 | 1.43 | 2.53 | 0 | 9 | 0.32 |
|  | Moderate-severe HY (3-4) | 7 | 1.71 | 2.06 | 0 | 6 |  |
| STIGMA | Mild HY (1-2-2.5) | 28 | 3.82 | 4.04 | 0 | 15 | 0.85 |
|  | Moderate-severe HY (3-4) | 7 | 3.71 | 3.82 | 0 | 11 |  |
| Item 32 Embarrassment | Mild HY (1-2-2.5) | 28 | 1.32 | 0.90 | 0 | 3 | 0.84 |
|  | Moderate-severe HY (3-4) | 7 | 1.43 | 0.79 | 1 | 3 |  |
| Item 33 Shame | Mild HY (1-2-2.5) | 28 | 0.57 | 0.10 | 0 | 3 | 0.82 |
|  | Moderate-severe HY (3-4) | 7 | 0.43 | 0.79 | 0 | 2 |  |
| TOTAL | Mild HY (1-2-2.5) | 28 | 23.21 | 19.63 | 1 | 84 | 0.34 |
|  | Moderate-severe HY (3-4) | 7 | 27.00 | 14.14 | 9 | 46 |  |

* Mann-Whitney

By PD phenotype (Pagano et al., 2016)

| SPARK | | N | Mean | SD | Min | Max | p* |
| --- | --- | --- | --- | --- | --- | --- | --- |
| PD SYMPTOMS | TD | 10 | 6.70 | 3.47 | 1 | 13 | 0.135 |
|  | AR | 24 | 10.83 | 7.70 | 1 | 30 |  |
| PHYSICAL | TD | 10 | 3.00 | 2.16 | 0 | 6 | 0.477 |
|  | AR | 24 | 2.67 | 2.60 | 0 | 9 |  |
| BODY IMAGE | TD | 10 | 2.60 | 2.41 | 0 | 8 | 0.313 |
|  | AR | 24 | 4.42 | 4.29 | 0 | 15 |  |
| SELF ESTEEM | TD | 10 | 1.20 | 2.78 | 0 | 9 | 0.349 |
|  | AR | 24 | 1.42 | 2.16 | 0 | 9 |  |
| STIGMA | TD | 10 | 2.80 | 2.74 | 0 | 8 | 0.552 |
|  | AR | 24 | 4.21 | 4.40 | 0 | 15 |  |
| Item 32 Embarrassment | TD | 10 | 1.60 | 0.70 | 1 | 3 | 0.241 |
|  | AR | 24 | 1.25 | 0.94 | 0 | 3 |  |
| Item 33 Shame | TD | 10 | 0.80 | 1.03 | 0 | 3 | 0.201 |
|  | AR | 24 | 0.46 | 0.93 | 0 | 3 |  |
| TOTAL | TD | 10 | 18.70 | 10.72 | 2 | 36 | 0.545 |
|  | AR | 24 | 25.25 | 20.69 | 1 | 84 |  |

* Mann-Whitney

TD: tremor-dominant; AR: akinetic-rigid; MIXED: mixed subtype.

PD patients were classified as either tremor-dominant (TD) or akinetic-rigid (AR) phenotype using the numerical ratio which was derived from patient's mean tremor score and mean akinetic-rigidity score. Tremor score included the following MDS-UPDRS part 3 items: resting tremor amplitude of the head and each limb (items 3.17a, 3.17b, 3.17c, 3.17d, 3.17e), constancy of rest tremor (item 3.18) as well as postural tremor of the hands (items 3.15a, 3.15b) and kinetic tremor of the hands (3.16a, 3.16b). The score was divided by 10, the number of single subitems included. The akinetic-rigidity score was the sum of the MDS-UPDRS part 3 following items: facial expression (item 3.2), passive range of motion rigidity of the neck and each extremity (items 3.3a, 3.3b, 3.3c, 3.3d, 3.3e), finger tapping (items 3.4a, 3.4b), hand movements (items 3.5a, 3.5b), pronation-supination movements of hands (items 3.6a, 3.6b), toe tapping (items 3.7a, 3.7b), leg agility (items 3.8a, 3.8b), arising from a chair (item 3.9), gait (item 3.10), freezing of gait (item 3.11), postural instability (item 3.12), posture (item 3.13), and global spontaneity of movement (item 3.14). The score was divided by 22, the number of single subitems included. Each item was rated 0–4 following the MDS-UPDRS scoring system. The mean of each scale was calculated and then the ratio (mean TD/mean AR score) determined. Patients were classified as AR subtype if they had a ratio < 0.8 or TD subtype if they had a ratio > 1.0 or mixed subtype if they had a value between 0.8 and 1. In addition, patients who had a positive mean in the numerator and a zero in the denominator were classified as TD. Patients with a zero in the numerator and a positive mean in the denominator were classified as AR.

Pagano, G., Ferrara, N., Brooks, D.J., and Pavese, N. (2016). Age at onset and Parkinson disease phenotype. *Neurology* 86(15)**,** 1400-1407. doi: 10.1212/WNL.0000000000002461.
