# Supplementary material for: Embarrassment and Shame in People With Parkinson's Disease: A New Tool for Self-Assessment
Source: Front Neurol. 2020 Jul 31;11:779. doi: 10.3389/fneur.2020.00779 (PMC7411180; doi:10.3389/fneur.2020.00779)
Supplement: Supplementary file 3 [file Table_3.DOCX]

**Supplementary data 3**

**Summary of patients’ comments obtained at the SPARK debriefing questionnaire**

Relevance: mainly related to the stage of the disease.

Missing questions: lack of questions on dysarthria, body posture, stiffness, REM-sleep behavior disorder and daytime somnolence. Change "excess of salivation" by trouble with saliva (for example, dryness of mouth). Differentiate on and off states. Context is also important (at home/outside home).

Embarrassing questions: item 20 (sphincter difficulties).

Difficult questions: items 6 (image of me that others perceives), 21 (losing body control), 26 (word “incompetent”), 29 (being taken seriously), 32 (embarrassed) and 33 (shame).

Other comments: changes in scoring system (example: “a little” difficult to differentiate from “moderately”; or: no-a little-a lot-enormously), identify the symptom which predominantly induces shame, differentiate shame in relation to myself and in relation to others. Add a column “Does not concern me”.

Items about dysarthria, body posture and stiffness were subsequently added. Due to the fact that only a single patient mentioned shame and embarrassment secondary to sleep-related disturbances such as sleepiness or REM-sleep behavior disorder and because we wished to keep the scale as short as possible to allow sufficient patient’s attention and feasibility, an item about sleep disturbances was not included in the final version of the scale.

**List of comments:**

Relevance:

- Some questions exceed the stage of my disease. I am not there yet!
- At the start of the disease, I felt a lot of embarrassment due to my tremor. Now, with the treatment I don't have embarrassment anymore because my tremor has disappeared. The feeling of embarrassment is not something that is fixed and established for good. With time I learned to avoid feeling embarrassed in avoiding the situations I know will make me feel embarrassed. The disease becomes a part of ourselves.
- I don't tell people that I have PD. People who don't know I have PD don't feel awkward with me. I don't know what I will feel when people will know that I have PD. My daughter does not understand for example.

Missing questions:

- Dysarthria is missing.
- Body posture is not addressed by SPARK. Problem with my posture is my main problem.
- Frequency of "feeling of shame" is not addressed by the questionnaire, only the intensity of the feeling.
- Embarrassment at restaurants is not addressed.
- Lack of the chronology: time before treatment, time after introduction of treatment. The feeling of shame was more important before introduction of treatment.
- Lack of the word "stiffness".
- Item 3 should be changed: "excess of salivation" should be replaced by trouble with saliva. The problem is not only drooling but also dryness of mouth that induces problem when I talk.
- I am a Commissioner of Police. I feel embarrassed by my tremor especially during formal/official events.
- Shouting during sleep and somnolence during the day are missing.
- A grading system out of 5 would be better; ON symptoms should be distinguished from OFF symptoms; I feel ashamed in my OFF state but I don’t feel ashamed during my ON stage. I feel ashamed of what I did during my ON stage but I feel embarrassed when I am not in my “super ON”. The feelings of shame and embarrassment are different depending if I am at home or outside. If I am at home, I don’t feel ashamed.

Embarrassing questions:

- Item 20 on sphincter incompetence.
- Questions are relevant and embarrassing but I expected it when I accepted to participate to the study.

Difficult questions:

- Item 21 (loss of body control): I find it hard to understand; item 26: word "incompetent" is not right: I feel as competent as before but I cannot express my competence because I feel tired and because I have concentration difficulties.
- Item 6 on deteriorated body image (image of me that others perceive and judge). I don’t know what people think of me.
- Item 29 on stigmatization, item 33 (shame).
- Item 32 (embarrassment).

Other comments:

- Ask what is the symptom which predominantly induces shame. For me: tremor and hypophonia.
- I feel that certain subjects mentioned here are not necessarily linked to PD but could pertain to age, gender or another illness.
- The rating scale "a little" does not seem different to "moderately".
- SPARK questionnaire should be done with a heath professional well known to the patient or able to put the patient at ease; dryness of mouth and not only drooling should be mentioned. Dryness of the mouth induces difficulties to express myself and I feel embarrassed by that.
- I suggest to change the rating scale: no, a little, a lot, enormously.
- Should be differentiated: shame in relation to myself and shame in relation to other's people eyes.
- Difference between people who understand the disease and people who don't understand the disease or don't want to understand the disease.
- It is important to be with a healthcare professional. It is important to not try to hide the truth.
- I feel rarely embarrassed. However, when I do, the feeling is very intense and I cope with difficulties, I lose my confidence. I would feel much more ashamed if I had cognitive difficulties.
- Item 20 (sphincter problems) should be in a specific item and not among stomach problems and sexual difficulties because it is my main problem (shame secondary to urinary urgencies).
- Clarify if shame is in relation to myself or to the others; add a column for "I don't experience this symptom".
- You should distinguish shame in relation to myself or in relation to others.
